# “I'm in pain and I want help”: An online survey investigating the experiences of tic-related pain and use of pain management techniques in people with tics and tic disorders

**DOI:** 10.3389/fpsyt.2022.914044

**Published:** 2022-08-05

**Authors:** Evangeline Taylor, Seonaid Anderson, E. Bethan Davies

**Affiliations:** ^1^Population and Lifespan Sciences, Queen's Medical Centre, School of Medicine, The University of Nottingham, Nottingham, United Kingdom; ^2^Neuro-diverse.org, Brussels, Belgium; ^3^NIHR MindTech MedTech Co-operative, School of Medicine, Institute of Mental Health, The University of Nottingham, Nottingham, United Kingdom; ^4^Clinical Neurosciences and Mental Health, School of Medicine, Institute of Mental Health, University of Nottingham, Nottingham, United Kingdom

**Keywords:** pain, pain management, quality of life, tic disorders, Tourette syndrome

## Abstract

**Objectives:**

Tic disorders (TDs) are complex neurological conditions characterized by involuntary, persistent vocalizations and motor movements called tics. Tics involve brief muscle movements and can impair many aspects of daily functioning and quality of life in patients – and their physical nature can cause pain. Understanding individuals' experiences of tic-related pain and pain management could help explore this under-researched area and identify additional support needs for this population. The aim of this study was to investigate experiences of pain and use of pain management techniques in people with tic disorders.

**Methods:**

An online survey consisting of multiple choice and open-ended questions exploring experiences of tic-related pain, help-seeking behavior for tic-related pain, and use of pain relief techniques for tic-related pain, was circulated online *via* international Tourette syndrome patient associations, and one online support group for Tourette syndrome. The online survey was open to adults (≥16 years) with self-reported tics. Open-ended questions were analyzed using thematic analysis.

**Results:**

One hundred eighty-one participants (16–71 years; 58.0% female) from 18 countries completed the online survey. Several aspects of tics were associated with pain, including the physical effort of motor tics (*n* = 177, 97.8%), repetitive tics (*n* = 141, 77.9%) and the consequences of tics (*n* = 131, 72.4%). Nearly two-thirds (*n* = 118, 64.6%) had sought professional help for tic-related pain. Distraction techniques (*n* = 126, 69.6%), taking pain relief medication (*n* = 125, 69.1%) and altering tics (*n* = 111, 61.3%) were the most commonly-reported methods used to relieve and cope with tic-related pain. Thematic analysis found an interrelated complex relationship between participants' tics, pain, and pain management techniques, reflected in four themes: the “tic-pain” cycle, the impact of pain, the importance of support, and the perceived successfulness of pain management techniques.

**Conclusions:**

Tic-related pain was reported to have a significant physical and psychological impact which impacted aspects of daily living in people with tic disorders. The findings add to limited research suggesting tic-related pain is a dominant issue for individuals with tic disorders, potentially impacting upon their quality of life. Increased understanding of tic-related pain and its influence may be helpful in the long-term management of tic disorders, both in terms of clinical management and patients' self-management.

## Introduction

Tic disorders (TDs)–such as Tourette syndrome (TS) and chronic motor or vocal tic disorder (CTD)–are complex neurological conditions characterized by tics: these are abrupt, involuntary, and persistent vocalizations and motor movements (1). Tics typically begin in early childhood, with TS having an ~1% global prevalence rate (1, 2). Motor tics involve a muscle or a group of muscles, which results in movements such as jerking of the neck or shoulders, eye blinking, or twitching. Vocal tics comprise of noises produced by the nose, mouth, or pharynx, such as whistling, throat clearing, and sniffing (3). Tics can be simple or complex in nature, with complex tics becoming more common with increasing age (4). Not all tics may be rapid and clonic: some tics may be dystonic and slower in nature and cause brief abnormal posture, while tonic tics involve brief muscle tensing and contractions (5). Options for treatment and management for tic disorders includes pharmacological medications and psychological interventions (e.g., behavioral therapy) (2, 6). Many individuals with tics also experience a premonitory urge–a physical feeling or sensation–before tics are expressed. Through awareness of this premonitory urge, tics can be suppressed–which many patients describe as uncomfortable (7). Tics are known to fluctuate or “wax and wane” in their frequency and severity over time (8), usually peaking in adolescence and improving in late adolescence-early adult years. The majority of people (85.7%) with TS have a comorbid psychiatric condition (9): attention deficit hyperactivity disorder (ADHD) is the most common, affecting between 60 and 80% of patients with TS (10).

There is strong evidence demonstrating that adults with tic disorders experience a lower quality of life (QoL) with resultant impairments across many aspects of their lives. Increased tic severity in adults has been associated with greater functional impairments and more life dissatisfaction, compared to the general population (11). Higher rates of clinical depression and depressive symptoms have been found in individuals with TS, compared to individuals without TS (12). Data from the Swedish National Patient Register found tic disorder patients have an increased risk of attempting and dying by suicide compared to the general population (13). The visible nature of tics means people with tic disorders often receive unwanted, negative attention from other people: this can lead to people suppressing their tics in social situations to avoid and cope with negative attention (14). Social stigma surrounding tics has led to adults with TS to report fears of discrimination and leading to feelings of isolation (15).

Given the exertion of muscles involved in tics, pain may be considered an “invisible” or unseen aspect of living with tic disorder. Riley and Lang (16) described patients with various tics that caused them pain, including neck pain from neck jerking tics, self-injurious tics (e.g., touching a hot stove), and headaches from full-body tics. An online survey with TS patients found 97% of participants reporting experiencing tic-related pain (17), while Conelea et al. (11) found that 60% of adults with tic disorders reported at least one tic that caused them pain or physical damage. A recent study found 60% of children with TS reported pain arising from tics, with increased pain significantly associated with greater tic severity (18). Additionally, higher rates of generalized joint hypermobility have been reported in people with neurodevelopmental conditions (including TS), with a relationship found between self-reported musculoskeletal pain and increased joint hypermobility (19). Adults with tic disorders have also reported a worsening in their tics and increased self-injurious behaviors during the COVID-19 pandemic (20).

Despite the clear evidence of pain associated with tics, many have identified a lack of research within this area (16, 17, 21, 22). In April 2021, the National Institute for Health and Care Excellence published guidelines about the assessment and management of all chronic pain (23). It has been suggested that the persistent pain experienced from tics and tic disorders falls into the chronic secondary pain category. However, these guidelines do not specifically include the management of pain where an underlying condition accounts for the pain, such as arising from a tic disorder. There is a concern that patients who are diagnosed with secondary pain may not be properly recognized nor treated appropriately (23).

The pain associated with tics often has lasting effects; 87% described physical discomfort from tics impacted upon their everyday life (17). Adults with TS-related headaches have been found to have poorer QoL and higher tic severity compared to children with TS and headaches (21). As well as coping with tics, people with tics are often reliant on themselves to find ways of managing and coping with tic-related pain. Pain caused by tics has been reported as an influential factor in deciding to commence treatment, such as medication or behavioral therapy (24). A study with children and young people with TS found that to cope with tic-related pain, younger children reported seeking support from their parents, while adolescents preferred to isolate themselves to cope (18). Due to complex assessment and treatment pathways, extremely long waiting times, and insufficient funding, accessing specialist care is often difficult to access for many patients with TS (22, 25). Anderson et al. (17) found 65% of participants reported they felt their tic-related injuries had not been effectively treated.

The need to explore the methods individuals with TS use to manage pain has been emphasized by previous research (26). Those with more severe tics may be more likely to use tobacco, alcohol, or illegal drugs to manage tics (11). Patients may not take medication for tics due to side effects (17, 25, 27), and so may use other methods to manage tics and tic-related pain. A variety of behavioral and cognitive self-management techniques for individuals with chronic pain conditions have been reported (28), including methods to improve an individual's ability to manage and cope with pain (e.g., relaxation, distraction techniques) (29, 30). However, much like research regarding tic-related pain, there is a lack of in-depth investigation into pain management among people with tics. Exploring this area could help to improve the knowledge of healthcare professionals and identify further areas of clinical need to be incorporated into clinical care for TD patients. Subsequently, this may improve the support available for individuals with TDs and their family members. The aim of the present study was to investigate experiences of tic-related pain and use of pain management techniques in people with TDs. This was explored through a mixture of quantitative and qualitative approaches *via* an online survey to explore people's lived experiences of tic-related pain, the methods people have used to manage tic-related pain, and exploring whether they have sought and received any support and/or treatment from healthcare professionals for tic-related pain.

## Materials and methods

### Participants and recruitment

Participants were adults (≥16 years) with a suspected or confirmed TD diagnosis, who were experiencing tics and tic-related pain at the time of participation. Based on previous online surveys using similar methodologies (11, 17, 31), we aimed to recruit between 80 and 150 participants. Participation was open to anyone worldwide who could read and write English.

Several text-based and image advertisements were created to promote the online survey. The advertisements contained the link to the online survey (hosted on JISC Online Surveys): the first two webpages presented full information regarding the study. Eight national TS charities were contacted by the first author (ET), and four agreed to disseminate the advertisements on their websites and social media pages. The advert was also posted to one online TS support community, on the NIHR MindTech MedTech Co-operative Twitter account, and the second author (SA) circulated it to forty-seven TS patient support associations. The survey was open between 10th June and 13th July 2021.

### Online survey design

An online survey as developed, comprising of multiple-choice and open-ended questions, taking ~30 min to complete. The first three webpages consisted of participant information (e.g., explaining study purpose, rights to withdraw, study ethical approval) and completion of an online consent form to indicate their willingness to participate. After consenting, the online survey consisted of six sections. The first section asked participants demographic questions about themselves. The second section asked participants about their tic disorder (e.g., whether they had a formal diagnosis, if they were currently prescribed medication for tics). An adapted version of the “interference” subsection of the self-report Brief Pain Inventory-Short Form (BPI-SF) (32) was used to assess perceived interference of tics. The interference subsection evaluates pain interference on seven aspects of daily life in the past week. This was adapted for the present study to ask participants about how much tics interfered on each of these seven aspects, with six additional aspects included based on tic disorder literature (11, 33). Each item was scored on a 1 (“No interference at all upon my daily life”) to 5 (“Severe interference at all upon my daily life”) scale, and included a “non-applicable” option. Average scores were calculated for each of the 13items.

The third section consisted of questions regarding participants' tic-related pain, including inviting participants to qualitatively share their experiences of tic-related pain, and the adapted BPI-SF focussing on tic-related pain interference in the past week. The fourth section presented the Brief Resilience Scale (BRS) (34): this consists of six statements measuring psychological resilience and ability to cope, each measured on a 1 (“strongly disagree”) to 5 (“strongly agree”) scale. Scores range between 6 and 30, with an average score calculated to indicate low (scores <2.99), normal (scores 3–4.30) and high (scores >4.31) psychological resilience (34).

The fifth section presented a multiple-choice question asking participants whether they had sought out professional help for tic-related pain, and if so they were asked to select from a list of 13 healthcare professionals/services with the option to specify other healthcare professionals/services. A second multiple-choice question asked participants to select–from a list of 17 active and passive pain management techniques (30)–which techniques/methods they had used for managing tic-related pain, or to specify any additional techniques they employed. Participants were invited to qualitatively share what they found helpful and unhelpful in managing tic-related pain. The final section presented assessed current tic severity through the Adult Tic Questionnaire (ATQ) (35), a self-report questionnaire assessing the presence, intensity and severity of 27 specific vocal and motor tics. Scores are summed to produce a total tic severity score (range 0–216), with higher scores indicating greater severity. The final page consisted of debriefing information alongside signposting to worldwide TS/tic organizations and the research team's contact details.

### Public involvement

Four adults with TS reviewed the online survey and advertisements prior to going live, to ensure they were worded appropriately and sensitively. The advertisements and questions were deemed appropriately worded, with minor adjustments made to some survey questions based on feedback.

### Ethical considerations

The study was reviewed and approved by the University of Nottingham Division of Rehabilitation, Aging and Wellbeing ethics committee.

### Data analysis

Data were downloaded into a Microsoft Excel spreadsheet, and quantitative data were analyzed descriptively in SPSS V26 (IBM Corp., Armonk, N.Y., USA). Relationships between resilience and tic severity were explored *via* Spearman's rank order correlation, with statistical significance set at *p* = <0.05. There was no missing data for the outcome measures. Responses to open-ended questions were analyzed using thematic analysis (36) by the first author (ET), using a data-driven inductive approach to analysis. The first stage involved familiarization through re-reading the responses and creating short codes summarizing the data. Secondly, similar codes were grouped together, leading into making connections between similar codes and collating these into potential themes. A thematic map was created to review the consistency and appropriateness of codes and themes, and discussed with BD for clarity and refinement. Theme names were generated, capturing the patterns in the data.

## Results

### Sample demographics

In total 181 participants (58.0% female, mean age 28.4 ± 12.2) years from 18 countries completed the survey (Table 1). The majority (*n* = 153, 84.5%) had a formal tic disorder diagnosis, experienced premonitory urges prior to their tics (*n* = 167, 92.3%, with over three-quarters (*n* = 144, 79.5%) reporting at least one co-morbid condition. The total average tic severity score from the ATQ was 71.59 (*SD* = 35.11, range = 9–193), with greater severity of motor tics (M = 45.49, SD = 18.62) reported compared to vocal tics (M = 26.09, SD = 19.16). Over half the sample reported their tics had become worse by “a lot” (*n* = 58, 32.0%) or “a little” (*n* = 47, 26.0%) since the start of the COVID-19 pandemic.

**Table 1:** Demographic characteristics of the sample (*N* = 181).

	***N* (%)**
**Gender**	
Male	58 (32.0%)
Female	105 (58.0%)
Non-binary	18 (10.0%)
**Age (M, SD)**	28.41 (12.19)
16–18 yrs	41 (22.7%)
19–25 yrs	58 (32.0%)
26–35 yrs	38 (21.0%)
36–45 yrs	24 (13.3%)
46–55 yrs	12 (6.6%)
56–65 yrs	6 (3.3%)
66–71 yrs	2 (1.1%)
**Country**	
United Kingdom	64 (35.4%)
Norway	41 (22.7%)
USA	23 (12.7%)
Australia	19 (10.5%)
Netherlands	10 (5.5%)
Canada	5 (2.8%)
New Zealand	5 (2.8%)
Argentina	2 (1.1%)
Belgium	2 (1.1%)
France	2 (1.1%)
Costa Rica	1 (0.6%)
Finland	1 (0.6%)
Germany	1 (0.6%)
Guatemala	1 (0.6%)
Ireland	1 (0.6%)
Spain	1 (0.6%)
Sweden	1 (0.6%)
Uruguay	1 (0.6%)
**Received TD diagnosis**	
Yes	153 (84.5%)
No	10 (5.5%)
Awaiting assessment	17 (9.4%)
**Self-reported co-morbidities**	
Anxiety disorder	94 (51.9%)
Depression	74 (40.9%)
ADHD	56 (30.9%)
OCD	49 (27.1%)
ASD	25 (13.8%)
Insomnia	24 (13.3%)
Learning difficulties	18 (9.9%)
SPD	18 (9.9%)
Dyspraxia	3 (1.7%)
Other	27 (14.9%)
None	37 (20.4%)
**Currently taking medication for TD**	
Yes	47 (26.0%)
No	134 (74.0%)
**Received behavioral therapy for TD**	
Yes	63 (34.8%)
No	111 (61.3%)
Unsure	7 (3.9%)
**Experiences premonitory urges**	
Yes	167 (92.3%)
No	8 (4.4%)
Unsure	6 (3.3%)
**ATQ total tic severity score (M, SD)**	71.59 (35.11)
ATQ motor tics severity scale (M, SD)	45.49 (18.62)
ATQ vocal tics severity scale (M, SD)	26.09 (19.16)
**Self-perceived change in tics since COVID-19 pandemic**	
Much better	4 (2.2%)
Little better	9 (5.0%)
No change	63 (34.8%)
Little worse	47 (26.0%)
Much worse	58 (32.0%)
**Self-reported condition causing pain (unrelated to tics)**	
Yes, diagnosed condition causing pain	33 (18.2%)
Yes, not diagnosed but have condition causing pain	11 (6.1%)
No	111 (61.3%)
Unsure	26 (14.4%)

Spearman's rank order correlation found a significant negative relationship between total tic severity and resilience scores [r_s_(181) = −0.154, *p* = 0.03], suggesting greater resilience was associated with less severe tics (Table 2). A Kruskal-Wallis test found a significant difference in total tic severity score by level of psychological resilience (low resilience, *n* = 105, M = 75.26 ± 35.95; normal resilience, *n* = 70, M = 63.36 ± 29.90; high resilience, *n* = 6, M = 103.33 ± 52.22), *H*(2) = 7.14, *p* = 0.028. *Post-hoc* Mann-Whitney tests found this significant difference was only between the low (md*n* = 72.0) and normal (md*n* = 58.5) resilience groups (*U*(n_low_ = 105, n_normal_ = 70) = 2982.00, *z* = −2.11, *p* = 0.035), suggesting those in the lower resilience group had greater median total tic severity.

**Table 2:** Total tic severity scores presented by resilience threshold.

**Brief resilience**	**ATQ motor Tic**	**ATQ vocal Tic**	**ATQ total tic**
**scale category**	**severity subscale (M, SD)**	**severity subscale (M, SD)**	**severity (M, SD)**
Low resilience (*n =* 105)	47.21 (19.27)	28.05 (19.05)	72.26 (35.95)
Normal resilience (*n =* 70)	41.63 (16.43)	21.73 (16.80)	63.36 (29.90)
High resilience (*n =* 6)	60.50 (22.23)	42.83 (33.07)	103.33 (52.22)

Looking at self-reported impact of tics on daily activities in the past week, the highest scores (out of 5) for interference were upon their academic studies (M = 3.63, SD = 1.16), self-esteem (M = 3.63, SD = 1.17), mood (M = 3.57, SD = 1.02), and sleep (M = 3.46, SD = 1.21) (Table 3).

**Table 3:** Self-reported interference of tics and tic-related pain across thirteen domains in the previous week.

**Domain**	**Impact of**	**Impact of tic-**
	**tics, M (SD)**	**related pain, M (SD)**
General activity	3.24 (1.03)	2.9 (1.21)
Mood	3.57 (1.02)	3.31 (1.24)
Walking ability	2.41 (1.19)	2.28 (1.41)
Typical daily work	3.13 (1.20)	2.73 (1.28)
Self-esteem	3.63 (1.17)	2.77 (1.41)
Family relationships	2.52 (1.32)	1.95 (1.23)
Relationships with friends	2.56 (1.30)	2.09 (1.23)
Relationship with partner	2.24 (1.34)	1.78 (1.15)
Social situations	3.86 (1.07)	2.74 (1.38)
School or education	3.63 (1.16)	2.78 (1.49)
Work/employment	3.23 (1.32)	2.72 (1.40)
Sleep	3.46 (1.31)	3.24 (1.40)
General enjoyment of life	3.09 (1.21)	3.04 (1.32)

*NB, Each domain is scored on 1-to-5 scale*.

### Experiences of pain related to tics

The most common types of pain were caused by the physical effort of motor tics (*n* = 177, 97.8%); repetitive tics (*n* = 141, 77.9%); and the consequences of tics (*n* = 131, 72.4%).While the primary goal of tic medication is upon tic expression, patients may experience subsequent changes in pain following changes in tic expression due to taking medication: of the *n* = 47 currently taking medication for their tics, almost half (*n* = 22, 46.8%) reported it made no difference to their tic-related pain, with a third (*n* = 17, 36.2%) reporting it helped relieve or manage tic-related pain. Likewise, of the *n* = 63 who had received behavioral therapy for tics, the majority (*n* = 35, 55.6%) stated it had no difference to tic-related pain, with *n* = 12 (19.0%) reporting it helped relieve or manage tic-related pain and *n* = 7 (11.1%) reporting it increased or intensified pain (Table 4).

**Table 4:** Self-reported causes of tic-related pain and impact of treatment upon tic-related pain.

	***N* (%)**
**Causes of tic-related pain**
Physical effort of motor tics (e.g., muscular pain, joint pain, cramping)	177 (97.8%)
Arising from repetitive tics (e.g., tendonitis, repetitive stress injury)	141 (77.9%)
Consequences of tics (e.g., injury due to tics)	131 (72.4%)
Physical effort of vocal tics (e.g., sore throat, sore nose)	120 (66.3%)
Pain from self-injurious tics (e.g., striking another object)	120 (66.3%)
Pain from suppressing tics	108 (59.7%)
Pain from premonitory urge	46 (25.4%)
Other	3 (1.7%)
**Has medication for tics helped tic-related pain? (*****n** **=*** **47)**
Yes - helped relieve or manage pain	17 (36.2%)
Yes - increased or intensified pain	1 (2.1%)
No, do not affect tic-related pain	22 (46.8%)
Not sure	7 (14.9%)
**Has behavioral therapy helped tic-related pain? (*****n** **=*** **63)**
Yes - helped relieve or manage pain	12 (19.0%)
Yes - increased or intensified pain	7 (11.1%)
No, do not affect tic-related pain	35 (55.6%)
Not sure	9 (14.3%)

Looking at self-reported impact of tic-related pain in the past week, the highest scores (out of 5) for interference of tic-related pain were upon their mood (M = 3.31, SD = 1.24), sleep (M = 3.24, SD = 1.40), and upon enjoyment of life (M = 3.04, SD = 1.32) (Table 3).

### Help-seeking and self-management for tic-related pain

Almost two-thirds (*n* = 118, 64.6%) reported seeking out professional help for tic-related pain, with over half of these (*n* = 71, 60.1%) reporting having received help/treatment. Of these, commonly-reported sources of professional help for tic-related pain included non-specialist doctors (*n* = 61, 51.3%), physiotherapists (*n* = 43, 35.9%) and neurologists (*n* = 40, 34.2%) (Table 5).

**Table 5:** Self-reported help-seeking from healthcare professionals for tic-related pain.

	***N* (%)**
**Help-seeking for tic-related pain**
Sought help and received help/treatment	71 (39.2%)
Sought help but did not receive help/treatment	46 (25.4%)
No help sought	61 (33.7%)
Not sure	3 (1.7%)
**Who did you seek professional help from? (*****n** **=*** **117)**
Non-specialist doctor (e.g., GP)	60 (51.3%)
Physiotherapist	42 (35.9%)
Neurologist	40 (34.2%)
Psychologist	29 (24.8%)
Psychiatrist	24 (20.5%)
Osteopath	14 (12.0%)
Behavioral therapist	12 (10.2%)
Pain management service/clinic	7 (6.0%)
Pediatrician	6 (5.1%)
Psychotherapist	6 (5.1%)
Other specialist doctor	6 (5.1%)
Nurse	4 (3.4%)
Occupational therapist	3 (2.5%)
Other^*^:
Child and adolescent mental health services	2 (1.7%)
Chiropractor	2 (1.7%)
Massage therapist	2 (1.7%)
Bowen therapist	1 (0.8%)
Endocrinologist	1 (0.8%)
Medical cannabis clinic	1 (0.8%)
Traumatologist	1 (0.8%)
**Taking prescribed pain relief medication for tic-related pain**
Yes, currently taking medication	13 (7.2%)
Yes, in the past	31 (17.1%)
No	134 (74.0%)

* *Responses under “Other” were from an optional text-box where participants could share further healthcare professionals/services they had sought help from*.

The most commonly-reported pain management techniques for tic-related pain were distraction tactics (*n* = 126, 69.6%), using over-the-counter/non-prescription medication (*n* = 125, 69.1%), attempting to alter tic that causes pain (*n* = 111, 61.3%) and using relaxation techniques (*n* = 107, 59.1%) (Table 6).

**Table 6:** Self-management techniques used to manage tic-related pain.

**Technique/method**	***N* (%)**
Used distraction tactics (e.g., watching videos, listening to music)	126 (69.6%)
Taken over-the-counter/non-prescription pain relief medication (e.g., tablets, medicated gels)	125 (69.1%)
Attempted to alter tic that causes pain (e.g., through suppression, redirection)	111 (61.3%)
Used relaxation techniques (e.g., breathing techniques)	107 (59.1%)
Used temperature-related treatment on site of muscle pain (e.g., heat or cold packs)	105 (58.0%)
Hands-on treatment (e.g., massage, acupuncture)	103 (56.9%)
Avoided certain activities which exacerbate tics and pain	88 (48.6%)
Exercises (e.g., stretching exercises)	86 (47.5%)
Used something to reduce tic impact (e.g., padded collar, gloves, brace)	80 (44.2%)
Sought out support from other people with tics	62 (33.7%)
Used psychological techniques (e.g., mindfulness, CBT)	50 (27.6%)
Used cannabidiol-based products	35 (19.3%)
Used cannabis	27 (14.9%)
Used electronic pain relief (e.g., TENS)	23 (12.7%)
Consumed alcohol	21 (11.6%)
Used elastic therapeutic/kinesiology tape	19 (10.5%)
Used drugs/substances	9 (5.0%)
Other^*^:	
Physical activity	10 (5.5%)
Sleeping well	5 (2.7%)
Applying pressure to affected area	3 (1.6%)
Botox injections for tics	2 (1.1%)
Hot baths	2 (1.1%)
Hot drinks to ease throat	2 (1.1%)
Tobacco	2 (1.1%)
Dietary changes	1 (0.5%)
Herbal-based medicine	1 (0.5%)
Humor to cope	1 (0.5%)
Oxytocin nasal spray	1 (0.5%)
Use of wheelchair	1 (0.5%)

* *Responses under “Other” were from an optional text-box where participants could share further pain management techniques/methods*.

### Qualitative analysis

Through analyzing the written responses, four themes were generated: “The tic-pain cycle,” “The impacts of pain,” “The importance of support,” and “Successfulness of pain management techniques.” These themes are multifaceted as they interlink and influence each other. For each cause (i.e., tic/pain/injury) there is an effect (i.e., pain/injury/action), which are dependent on an individual's experience of tics, pain, and support. For ease of clarification, the themes have been separated into subthemes and appropriate links are discussed in relevant sections. From the qualitative data, it was clear that participants had an informed insight regarding their tics and were highly aware on how it impacted on themselves and others around them.

#### Theme 1: The tic-pain cycle

**Figure 1:**
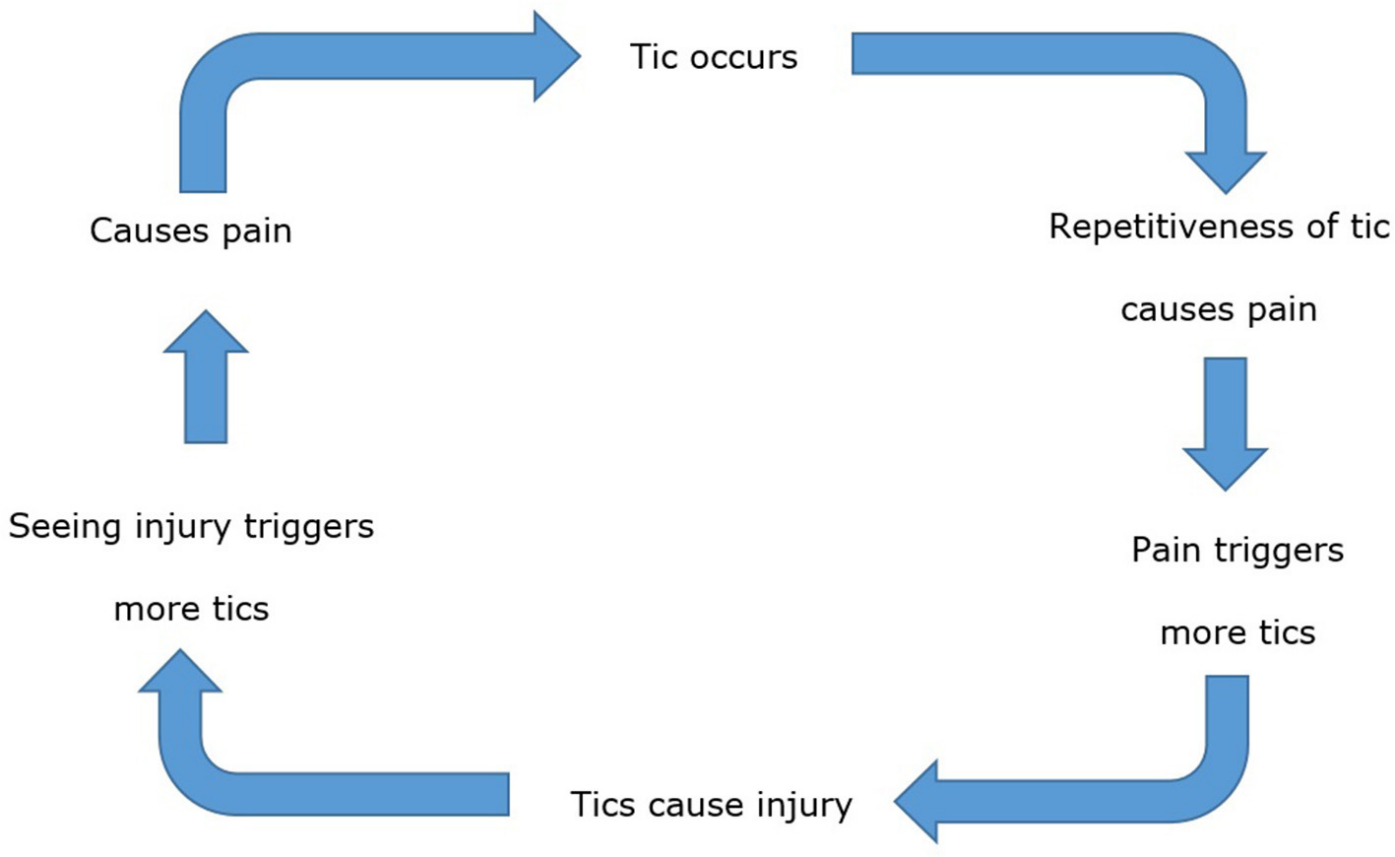
Theorized diagram of the “Tic-Pain Cycle.”

Although experiences differed, a reinforcement pattern between participants' tics and pain emerged; the repetitiveness of a tic was the main aggravator of pain, and this pain could then trigger more tics: “*I often go to bed with aches and pains and the more pain I'm in the more I tic”* (Participant 66, aged 25) (Figure 1). Consequently, tic-induced injuries could occur, and viewing or acknowledging these injuries could again cause tics: “*Seeing the bruises sets the hitting tics off”* (P134, aged 16). Aggravating these injuries caused further pain, prompting more tics: “*The pain was becoming constant … It was also making my tics worse and more painful themselves”* (P118, aged 17). This suggests reducing or improving the tics may be the most important thing to focus on in tic management.

However, it was unclear whether the steps within this cycle happened immediately after one another, or whether they occurred over a longer period of time. Some participants acknowledged a certain tic could be triggered within this cycle leading to its continuation: “*the pain of the bruises does not stop the tics, so I basically re-bruise it every day”* (P124, aged 17) – but others were vaguer in their description of any patterns. The emotional and mental state of participants also appeared to influence the tic-pain cycle, as many noted their tics and tic-related pain were exacerbated by heightened emotions: “*the pain ntenses [sic] when i have more tics. and that mostly happens if i have a lot of stress, if i am nervus [sic], when i am tired”* (P32, aged 27). These emotions seemed to continue to impact and/or cause tics and subsequently cause further pain: “*Stress reduction also reduces tics, which in turn reduces tic related pain”* (P89, aged 18).

Some participants discussed effects on the pain by attempting to suppress their tics. Many noted that supressing tics made them worse (e.g., increased intensity or frequency), subsequently increasing pain: “*suppressing my tics …. makes them worse in the long run which makes the pain worse too”* (P116, aged 19).

One participant acknowledged the importance of treating tics: “*there is no real solution until tics can be treated/cured”* (P128, aged 22). However, many participants noted that the pain - not the tics - was the reason for seeking help, suggesting this relationship is complex.

#### Theme 2: The impacts of pain

This theme describes how the tic-related pain affected participants psychosocially and physically. These two factors could be responsible in supporting decisions around help-seeking.

##### Psychosocial impact of pain

Two main patterns of psychological impact were described by participants, reflecting *hopelessness* and *acceptance*. For many, their outlook on living with tic-related pain was bleak, as many were not able to “*cope with the constant pain related to [their] tics”* (P26, aged 49). Some participants noted feelings of anger: “*it hurts all over and it makes me angry and i cry”* (P81, aged 39). Many described the pain as unbearable, and some were led to suicidal thoughts, emphasizing the serious and adverse impacts of tic-related pain and highlighting the desperation this group of patients are experiencing.

The interference of pain and its impact on daily life in some cases led to some individuals reaching out for support. For example, one participant explained they sought help as: “*chronic pain had me suicidal”* (P173, aged 35). Feelings of desperation, anguish and low mood, caused by pain were commonly acknowledged among participants. However, the aspect of hopelessness in terms of finding relief from pain also discouraged others from seeking help: “*I have never sought help because I didn't believe there was anything anyone could do to help with my particular problems”* (P59, aged 66). These indicate participants' low expectations concerning successful support and treatment.

Inversely, some participants exhibited feelings of acceptance surrounding tic-related pain. This was dependent on participants' experiences of tics and pain, with this appearing more common in those who perceived their pain as less severe and/or more tolerable: “*The pain is fairly constant, just in the background”* (P118, aged 17). Some individuals expressed a need to be proactive in managing their tic-related pain: “*I wanted to be able to have more control and possibily [sic] less tics, and so: less pain”* (P22, aged 30). Participants with this viewpoint appeared to have a more educated insight into the benefits of being self-sufficient in dealing with their pain: “*To find support and ways, to learn to prevent problems on the long term, and relieve on short term”* (P28, aged 46). However, other participants rejected seeking treatment for pain as their own methods of self-management were sufficient: “*At the end of the day its nothing a panadol or an Advil can't fix”* (P11, aged 17). This demonstrates a variety of experiences as, for some, tic-related pain can be less severe and more manageable.

##### Physical impact of pain

This subtheme encompasses the physical side effects and injuries from tics and pain, and how these affected participants' ability to seek help. Within the open-ended questions, the most reported symptoms were headaches (*n* = 55), with general muscle and throat soreness also frequently described. Bruises and damage to teeth were the most frequent injuries. Other less common consequences, were arthritis, broken bones, and cauliflower ear. The length and severity of tic-related pain and injuries was dependent on the type of tic specific to the individual, and reflects the diversity of the lived experiences of people with tics and TDs.

Participants' decision to seek help appeared to be affected by their outlook regarding their injuries. For some, acquiring medical help seemed obvious*: “I'm in pain and I want help”* (P66, aged 25). Similar to the psychosocial impacts of tic-related pain, many participants called the pain unmanageable, suggesting their methods of self-management were unsuccessful. Some revealed the potential danger their tics could create: “*My self-injurious tics were very severe … I was scared I would seriously hrt [sic] myself”* (P172 aged 19). These feelings of distress and worry relating to injury were frequent, again emphasizing the emotional impact. Many also participants stated their tic-related pain “*was so intense and disabling in day-to-day life”* (P107, aged 23), highlighting the seriousness of its impact upon daily living.

The physical consequences from tics and pain again elicited feelings of hopelessness when potentially seeking out support. Many believed this was pointless: “*What are they gonna do? Prescribe me otc [over-the-counter] pain meds? I dont think it would be of much use and I don't want to annoy my doctors”* (P128, aged 22). Participants may have previously had their experiences belittled by healthcare professionals. This is important as it accentuates past negative encounters adversely affect individuals and discourage them from pursuing help. Furthermore, this signifies that many are silently suffering. However, on the other hand, the physical impacts of tics and tic-related pain prompted some participants to seek help. Many reported receiving help from healthcare professionals, such as receiving massages for sore muscles or Botox injections, to help reduce the frequency of their tics and resulting tic-related pain.

#### Theme 3: The importance of support

This theme encompasses how various types of support effected participants' thoughts, feelings, and actions, with three generated subthemes: “failings of healthcare professionals,” “inaccessibility of treatment,” and “the balance of social support.”

##### Failings of healthcare professionals

Participants reported an unmistakable lack of understanding and empathy from healthcare professionals regarding their tics and related pain. Numerous participants described receiving inadequate care after seeking professional help: many reported their healthcare professionals were uneducated regarding TDs, dealing with tic-related pain, or dismissed their problems. One participant with spinal and rib tic-related injuries described their encounters with doctors: “*I've kind of just been told to deal with it, because I can't ‘stay still', which was the recovery advice for the fractured ribs too … a fully qualified GP … asked me what tourettes was, and said ‘is that a form of exercise?”'* (P8, aged 22).

Furthermore, many participants were aware of the societal stigma surrounding TDs, which led them to avoid seeking help due to potential embarrassment. One individual stated they had not sought treatment for tic-related pain as they were “*not sure how seriously I would be taken”* (P27, aged 49). Feelings of frustration and hopelessness were again extremely common relating to the failings of healthcare professionals: “*the health system where I live is not willing to help me because my issues are too complex. I have been refused or ignored at every turn”* (P145, aged 33). One participant noted they had “*lost faith in doctors”* (P155, aged 30) due to the difficulty in receiving a TD diagnosis and subsequent treatment. Experiences such as these provide further evidence to why participants may feel hopeless and reluctant to pursue help – healthcare professionals' insufficient TD knowledge appears to negatively impact on their ability to provide treatment.

##### Inaccessibility of treatment

Another common issue highlighting the importance of healthcare support was difficulties in access. Many participants described how both tic and pain management treatments were costly and unaffordable, with long waiting times to see healthcare professionals. These contributed to feelings of disheartenment and lack of confidence in the healthcare system, leading some to feel discouraged in seeking further support. Due to not having knowledge about available support, others reported being unaware of where to search for help. The inaccessibility of support again led to feelings of hopelessness regarding participants' ability to deal with their tics and pain, further highlighting the importance of effective help on their wellbeing.

##### The balance of social support

This subtheme refers to the physical and emotional assistance some participants received from family and friends. In general, they were sympathetic toward participants regarding their tic-related pain. Many participants described family members providing massaging or applying topical treatments to affected body parts: “*my legs would hurt … My mom would massage them for me”* (P33, aged 21). In some cases, this would replace the need to see healthcare professionals. Emotional support was also instrumental in encouraging participants to seek treatment: “*My wife forced me to seeking [sic] help. It was a time when the pain was insanely painful and I had thoughts of ending my life”* (P181, aged 41). However, some participants were negatively affected by perceived excessive social support: “*My family and friends wrap me in 'cotton wool' because of the fear associated with hurting me. They don't give me hugs for fear of crushing me … This causes a feeling of uselessness”* (P61, aged 23). This demonstrates the complexities and intricacies of social support, with an apparent fine line between providing an adequate amount and too much support.

Conversely, a small number of participants described a less supportive social network. Some participants described their friends and family having a lack of understanding regarding their tics and tic-related pain: “*friends and family never supported my tics, probably because they don't have them”* (P16, aged 34). Another participant felt deterred from seeking support from their parents concerning their tic-related pain as “*they seemed to be judgemental the first time I told them”* (P129, aged 21). Similarly, feelings of shame due to the stigma about tics prevented some participants for seeking out for support from family and friends: “*[a] doctor diagnosed me with tics but I was too ashamed and embarrassed to tell my parents about every tic I experienced”* (P128, aged 22). The apparent lack of understanding within social networks indicates issues around health beliefs and education regarding TDs and tic-related pain.

#### Theme 4: Successfulness of pain management techniques

Within participants' written responses, the pain management techniques that were the most notable in their varying success were medication, distraction and relaxation methods, and massaging of the affected muscles. Although medication was reported as most-used pain management technique, there was a diverse opinion of its usefulness. Specifically, it was clear participants felt it was both the most and least useful method of dealing tic-related pain. For some, the ease of access and successfulness of over-the-counter pain relief led them to acknowledge it positively: “*[Ibuprofen was] a godsend, you take 2 and 15 min later adios pain … for that brief moment, pure bliss”* (P11, aged 17). However, the obvious short-term impact of over-the-counter medication was unhelpful for others: “*painkillers … they take the edge off temporarily but it doesn't stop the pain”* (P163, aged 33).

Experiences also varied in the perceived usefulness of prescribed medication for tic and pain relief: “*medication that makes me tic less [relieves tic-related-pain]. Otherwise, idk, I take a lot advil”* (P137, aged 21). However, other participants felt the problematic side effects and ineffectiveness of taking long-term medication interfered with their ability to help manage pain: “*as soon as they wear off the bruises, burns, and other injuries hurt again. They can take weeks to fully heal, and its not safe to be on pain meds the whole time”* (P152, aged 30). While medication is an established treatment option for tics, it is not always the best for each individual.

Techniques that distracted or engaged participants' minds – including breathing techniques, mindfulness, meditation, watching videos, listening to music, and using stim toys - had varying degrees of successfulness in managing pain. These were often viewed as successful for two reasons. Firstly was whether this was used in combination with other pain relief methods: “*[breathing and stretching techniques] distracts myself and stop [sic] the tic, after the tic stops I try to stretch the area that is in pain to relieve the tensed muscles”* (P14, aged 34). Secondly, the distraction or relaxation methods were perceived as needing to sufficiently mentally engaging in order for participants to feel they are able to help them relieve tic-related pain: “*then I don't think about my tics and make them worse”* (P134, aged 16). On the other hand, for some participants relaxation techniques were noted to be very difficult for them to perform successfully: “*mindfulness, specifically mediation [sic]. I … struggle when I'm under stimulated … I end up thinking about my tics in lieu of anything else to focus on, which makes them worse”* (P134, aged 16).

Various forms of massages - including sports massage, physical/physiotherapy, and osteopathy - were noted to be the most helpful techniques to relieve pain: “*[they] address the muscle knots and build up of tension due to my tics”* (P15, aged 27). However, as evident within the theme *Inaccessibility of treatment*, these were often noted as expensive: “*financially difficult to do regularly and no NHS help”* (P45, aged 40).

## Discussion

The present study aimed to investigate the experiences of pain and use of pain management techniques in individuals with TDs and tics. To our knowledge, it is one of the first studies to focus on and explore the topic of pain in TDs, and adds to the limited literature investigating pain associated with tic disorders. The findings provide a valuable insight into the experiences of tic-related pain and methods used by this group to attempt to manage and alleviate such pain. Much like symptomology of tics, the interrelated and multifaceted aspects of tic-related pain and pain management illustrate a wide range of experiences.

As anticipated, the physical effort of motor tics – such as muscular and joint pain, and soreness - was the most endorsed cause of tic-related pain, consistent with previous literature (16, 37, 38). Previous research has reported that painful tics are one major factor in decisions to seek out treatment for tics (24); of the participants currently taking tic medication, a third stated medication had helped manage tic-related pain, but almost half reported no impact or change. This same sentiment was echoed throughout the qualitative data. While the primary aim of tic medication is upon tic expression, it can be anticipated to impact on tic-related pain too through modifying tic expression. Many participants noted using medication as a method of pain relief, yet it was commonly described as ineffective. The evidence for the effect of medication upon decreasing tic severity varies by type of medication and are often accompanied by side effects (39), which may impact upon patients' QoL but also the adherence and decisions made about medication (25). Participants' reported using a variety of active and passive behavioral, cognitive, pharmaceutical and medical methods for managing tic-related pain, aligning with self-management strategies previously reported in an Australian sample experiencing chronic pain (30). Something of note here is participants' use of strategies to alter tics causing pain - such as through suppression or re-direction – and these strategies may themselves be painful, as over half mentioned they experienced pain through suppressing their tics. Again, this notion was reinforced within the written answers from participants. Compared to matched controls, people with tic disorders have an increased risk of attempting and dying by suicide compared to a general population sample (13). It could be suggested that tic-related pain could play an important part in further impacting upon the mental health and QoL in individuals with tic disorders. This would seem to be an important aspect to think about in clinical care.

Similar to research conducted by Anderson et al. (17), while over a third reported seeking out and receiving professional help, a quarter had sought out help but not received it – possibly for various reasons. From the qualitative data, participants reported a lack of empathy and knowledge about tics from healthcare professionals, which appeared to impact their decisions and willingness to seek treatment. Healthcare professionals' lack of understanding about tic disorders and inadequate treatment and management pathways for tics have been highlighted by people with tics and their families (15, 25, 31, 40, 41). While educating healthcare professionals about tic disorders and management pathways may help them provide treatment and improve the QoL of TD patients, at the same time the limited treatment options for tic disorder patients and difficulties accessing specialist help - as well as participants' reports of the complex relationship between tics and tic-related pain - may make it difficult to provide sufficient treatment. The NICE pain guidelines (23) advise that when assessing all types of chronic pain, clinicians should take a person-centered approach to identify factors contributing to the pain and how the pain affects the person's life. A positive development is that the recommendations suggest individualized assessment of patients in pain and for shared decision-making with the patient. There is the potential to include pain assessment and treatment for people with painful tics and encourage healthcare professionals to be aware of and use the NICE chronic pain guidelines for patients with tic disorders and painful tics.

The inaccessibility of treatment may have also influenced participants' abilities and preferences for seeking and receiving professional help. As noted in previous research (22, 25), many participants reported that the long waiting times and high cost of seeing healthcare professionals prevented them from accessing regular support for their tic-related pain. A recent international survey found a lack of specialized tic disorder clinics within the UK, USA, Canada, and Europe (42). This has important implications: if individuals cannot receive help for their tics, they will be unable to obtain support for the consequential pain. Therefore, access to evidence-based treatment and support is vital for the mental, emotional, and physical impacts of tics and other impactful aspects of living with tics – such as pain – that further impact on patients' QoL.

The repetitive nature of tics caused pain for many, leading to a theorized repetitive “tic-pain” cycle. Similar cyclical patterns have been reported among individuals with TS. One example are tic attacks (sudden bouts of tics and tic-like behaviors, lasting several minutes or hours): these have been described as a “vicious cycle” created by disproportionate attention toward a combination of physical sensations, cognitive elements, and anxiety-related beliefs (43), resulting in increased tic frequency and anxiety symptoms, which trigger further tic attacks. Similarly, tics and premonitory urges are known to increase due to stressful contextual triggers (44). Although the specific cognitive processes were not explored in the present study, the “tic-pain” cycle appears to contain similar features. As highlighted by the written responses in the present study, physical sensations experienced from tics, and emotional reactions from pain such as anger, frustration, and anxiety, can prompt further tics (45). The combination of these psychological and environmental factors may initiate and reinforce the “tic-pain cycle” - and it is evident that this relationship is complex. While responses on the adapted interference scale suggested that tics had a greater interference on participants' lives, qualitative responses suggested the pain from tics was more problematic than tics for some participants. Further investigation is required to understand this potential “tic-pain cycle.”

Overall, participants' free-text responses indicated how much tic-related pain impacted on their emotional wellbeing: feelings of hopelessness, desperation, and suicidal thoughts due to tic-related pain were reported. Participants with greater tic severity were more likely to have low psychological resilience scores, compared to those who screened within the “normal” range. Emotional functioning and resilience have been identified as important aspects of QoL in patient populations (33, 46, 47), and understandably tic severity can have greater impact upon individuals and their ability to cope with stressful events. Heightened levels of depression have been commonly reported among individuals with tic disorders and chronic pain (33, 48, 49). Additionally, decreased resilience is associated with greater depression (50) and greater disability among patients with chronic pain (46)-specifically among middle-aged and younger individuals (51). Treatment and management of tics should go beyond addressing tic frequency and severity, and expand onto other aspects contributing to QoL – such as ability to cope and resiliency (52), as well as the chronic secondary pain experienced from tics.

The present study also adds to the limited research investigating the physical impacts of tics, and highlights the enduring effects of tic-related pain. Through the free-text comments, headaches were commonly self-reported among participants, similar to recent international research (21, 53). Other more serious injuries due to tics were also reported, such as broken and fractured bones, slipped spinal disk, and arthritis. Self-injurious tics have been linked to an increased risk of TS patients developing a traumatic brain injury (54). These acute consequences indicate a need for treatment to be available to help those affected by tic-related pain and injuries.

From both the quantitative and open-ended data, the most reported pain management techniques were distraction, over the counter medication, and various hands-on treatment, such as massages. Many participants noted a difficulty in using mentally-stimulating distraction methods, such as breathing techniques, mindfulness, or meditation. As these methods require significant concentration and attention, it may be that tics themselves interrupt ability to practice these pain-management methods, and symptoms of co-occurring conditions (e.g., ADHD) may also hinder implementation – meaning that other pain-management techniques may need to be used. Distraction methods involving listening to music and playing games have been used with varying effectiveness in helping individuals with chronic pain cope and improve their QoL (55–58). A small pilot study found dancing along to music videos can be successful in helping reduce tic severity (59). Additionally, music has been found to help increase individuals with ADHD abilities to focus and sustain attention (60). Given the high comorbidity between TD and ADHD (10), this could be of use. Furthermore, as indicated by the “tic-pain” cycle, improvement of tics may help individuals to reduce pain, or assist those with pain to cope better. Therefore, future research could investigate the effectiveness of distraction methods that incorporate both music and exercise. In terms of clinical guidance for patients with tics who are experiencing pain, what is needed are clear pathways for patients to access pharmacological, psychological, physical therapy, exercise, acupuncture, electrical physical modalities, self-management and pain management programmes for their painful tics.

### Limitations

The present study did not aim to identify the prevalence of tic-related pain in individuals with TDs, and only captured participants' experiences at one point in time. Given that the study was conducted online, individuals without internet access, or those who did not visit the social media pages and websites where the study was advertised, would not have seen the advertisement and therefore not accessed the online survey. Participant attrition may arise from the longer length of online surveys (such as in the present study), potentially meaning individuals with TS and comorbid ADHD may be under-represented (11), and so the results may not be fully generalisable to all people with TDs. Furthermore, although recruitment was through TS organizations across several different countries, the perspectives of those who could not respond in English was not collected. We also did not measure participants' satisfaction or appraisal of professional help they had sought out for their tic-related pain. Finally, the gender skew – in that more females with TDs participated in the present study - is noticeable and does not typically align with trends in TD literature reporting greater prevalence in males (61). There are well-known sex and gender disparities in the biopsychosocial experience of pain, and treatment and management of pain (62): findings from 13 European countries report greater prevalence of pain in women than men (63), and our sample could potentially reflect this. Previous research has found that compared to females, males with TDs were 1.78 times more likely to report tics resulting in pain (64). In finding a similar gender skew in their online survey with adults with TDs, Conelea and colleagues (11) speculate that this could be for several reasons including response bias in females being more likely to participate in research, and that it could reflect greater prevalence of TDs in adulthood in women compared to men.

## Conclusions

The present study is one of the first to conduct an in-depth exploration of pain and pain management techniques in individuals with tics and TDs. These results suggest tic-related pain is a complex and widespread issue, emphasizing a need for research into the impacts of pain on the QoL within this population. Significant work is still required to better equip both medical providers and the healthcare system to help patients with tics and pain. Evaluation regarding the effectiveness of pain relief methods is another area to be explored. By further investigating these matters, we can help to create and promote improved patient care for this population. The current lack of any NICE guidelines for tics and TDs continues to add to the difficulties to access not just treatment and management of TDs, but also in turn any recommended treatment or management of the pain associated with this condition.

## Data availability statement

The raw data supporting the conclusions of this article will be made available by the authors, without undue reservation.

## Ethics statement

The studies involving human participants were reviewed and approved by University of Nottingham Division of Rehabilitation, Aging and Wellbeing Ethics Committee. Written informed consent from the participants' legal guardian/next of kin was not required to participate in this study in accordance with the national legislation and the institutional requirements.

## Author contributions

ET, SA, and ED contributed to the conception and design of the study and wrote sections of the manuscript. ET and SA led on participant recruitment. ED performed quantitative analysis. ET led on qualitative analysis with support from ED. ET wrote the first draft of the manuscript. All authors contributed to manuscript revision, read, and approved the submitted version.

## Funding

This work was co-funded by the National Institute for Health (NIHR) MindTech Medtech Cooperative and the NIHR Nottingham Biomedical Research Centre.

## Conflict of interest

The authors declare that the research was conducted in the absence of any commercial or financial relationships that could be construed as a potential conflict of interest.

## Publisher's note

All claims expressed in this article are solely those of the authors and do not necessarily represent those of their affiliated organizations, or those of the publisher, the editors and the reviewers. Any product that may be evaluated in this article, or claim that may be made by its manufacturer, is not guaranteed or endorsed by the publisher.

## Author disclaimer

The views expressed are those of the author(s) and not necessarily those of the NHS, the NIHR or the Department of Health and Social Care.

## Abbreviations

ADHD: Attention deficit hyperactivity disorder
CTD: Chronic motor or vocal tic disorder
QoL: Quality of Life
TD/TDs: Tic disorder/Tic disorders
TS: Tourette syndrome.
